# Detection of Cervical Cancer Cells in Whole Slide Images Using Deformable and Global Context Aware Faster RCNN-FPN

**DOI:** 10.3390/curroncol28050307

**Published:** 2021-09-16

**Authors:** Xia Li, Zhenhao Xu, Xi Shen, Yongxia Zhou, Binggang Xiao, Tie-Qiang Li

**Affiliations:** 1 Institute of Information Engineering, China Jiliang University, Hangzhou 310018, China; sdlixia@126.com (X.L.); Zhenhao.Xu@cjlu.edu.cn (Z.X.); xi.shen@cjlu.cn.se (X.S.); Yongxia.Zhou@cjlu.edu.cn (Y.Z.); Binggang.Xiao@cjlu.edu.cn (B.X.); 2 Department of Clinical Science, Intervention and Technology, Karolinska Institutet, S-17177 Stockholm, Sweden; 3 Department of Medical Radiation and Nuclear Medicine, Karolinska University Hospital, S-14186 Stockholm, Sweden

**Keywords:** cervical cancer, Pap smear test, whole slide image (WSI), feature pyramid network (FPN), global context aware (GCA), region based convolutional neural networks (R-CNN), region proposal network (RPN)

## Abstract

Cervical cancer is a worldwide public health problem with a high rate of illness and mortality among women. In this study, we proposed a novel framework based on Faster RCNN-FPN architecture for the detection of abnormal cervical cells in cytology images from a cancer screening test. We extended the Faster RCNN-FPN model by infusing deformable convolution layers into the feature pyramid network (FPN) to improve scalability. Furthermore, we introduced a global contextual aware module alongside the Region Proposal Network (RPN) to enhance the spatial correlation between the background and the foreground. Extensive experimentations with the proposed deformable and global context aware (DGCA) RCNN were carried out using the cervical image dataset of “Digital Human Body” Vision Challenge from the Alibaba Cloud TianChi Company. Performance evaluation based on the mean average precision (mAP) and receiver operating characteristic (ROC) curve has demonstrated considerable advantages of the proposed framework. Particularly, when combined with tagging of the negative image samples using traditional computer-vision techniques, 6–9% increase in mAP has been achieved. The proposed DGCA-RCNN model has potential to become a clinically useful AI tool for automated detection of cervical cancer cells in whole slide images of Pap smear.

## 1. Introduction

Cervical cancer is the second most common malignancy among women with more than half a million new cases reported annually. It can be detected and prevented at its early stage by cytology screening test. Over the last six decades, cervical smear of Pap strains has played an important role in controlling cervical cancer epidemic by detecting pre-cancerous changes and providing guidance for early treatments. It was estimated that a Pap smear test can reduce mortality rate by 70% or more [1]. Cytological slides of vaginal smear of Pap strains are microscopically examined at 400× magnification. With such magnification, cytologists must examine thousands of field-of-views (FOVs) per slide and, thereby, are limited to investigate a limited number of samples per day. Despite the seemingly simplicity in detection of abnormal cervical cells, experienced physicians are required for an accurate diagnosis and there is a lack of such healthcare resources in many developing countries. Moreover, even with experienced pathologists, it is a tedious task to examine the Pap smear slides through microscope and the detection of cervical cancer cells can often be missed, due to the small size of the cervical intraepithelial neoplasia, overlapping clump of cells or masking by blood mucus and artifacts.

In the light of these challenges, different computer-aided diagnosis technologies have been proposed [2,3,4,5] to reduce the workload of pathologists and improve the efficiency and accuracy of cervical cancer detection. The computer-assisted screening for cervical cancer cells involves typically two steps: segmentation [6] of cytoplasm and nucleus, and classification [7]. After the entire image is segmented into nuclei and cytoplasm, these small pieces are classified into abnormal and normal ones, and finally used for the diagnosis of cervical cancer. The segmentation quality is, therefore, vital for the extraction of cell features and the consequential classification results. This has been a subject of extensive investigation [6,8,9,10,11,12,13,14,15] in the last two decades. Cell overlapping is one of the difficult issues for accurate cell segmentation and effective methods to separate overlapping cells have been proposed [11,16,17,18]. After successful segmentation into individual cells, the performance for cell classification relies on the extraction of morphological, structural, and contextual features and optimal selection of classifiers [19]. Due to the complicated characteristics of cervical cells and pathological subtleties associated with cervical cancer development, traditional computer vision methods can only provide limited generality and efficiency for segmentation and feature extraction. 

With the advent of deep learning based artificial intelligence (AI) technology in recent years, particularly, the development of convolutional neural networks (CNNs) has demonstrated its potential for improving automated cervical cancer screening and diagnosis. Deep learning excels at recognizing patterns and high-level sematic features in large volumes of data, extracting relationships between complex features in the images, and identifying characteristics that is difficult be perceived by the human brain. AI software can process vast number of images rapidly and has already been used to assist clinicians in level classification of skin cancer [20], breast cancer detection [21,22], end-to-end lung cancer screening [23] and prediction of colorectal cancer outcomes [24]. Significant progress has also been made in automated cervical cancer classification. With these classification methods we can leverage the deep CNNs for effective extraction of deep and high-level features [3,7,25,26,27,28,29,30,31] of the cells to achieve more accurate cancer screening. 

Deep learning technology has also led to breakthroughs in object detection, which refers to the task of both identifying the locations and categorizing object instances in images. Object detection is a key ability required by most computer and robot vision systems. The cutting-edge research in the field has been making amazing progress in many directions, such as automatic car-driving and object tracking. Current object detection models can be divided into two main categories, proposal-driven two-stage detectors and proposal-free single stage detectors. For the two-stage detectors, such as faster region-based CNN (FRCNN) series [32,33,34,35], region-based fully convolution network (R-FCN) [36] and feature pyramid network (FPN) [33], the detection process is completed in two steps. First the algorithms produce sparse candidate proposals, and then further refine the coordinates and classify these candidate proposals. The two-stage methods generally have higher accuracy at the cost of speed. In contrast, the one-stage detectors generate the object’s category labels and locations directly and have advantage in speed, such as YOLO [37] for real-time object detection, single shot multibox detector (SSD) series [38] and RetinaNet [39]. More recently, a new category of anchor-free single-stage detectors has emerged [40], which has completely abandoned the anchor mechanism.

The object detection methods have also begun to find applications for cervical cancer detection in cervical cytology images. At present, there are few network architectures designed specifically for the specific task of cervical cancer detection [41,42,43,44,45,46]. Xu et al. [45] used the generic Faster RCNN for the detection of abnormal cells in cervical smear images scanned at 20× and showed that detection of various abnormal cells was feasible. Zhang et al. [46] tested an R-FCN model for cervical cancer screening of liquid-based cytology (LBC) images. The performance evaluation was based on an interesting concept called hit degree which ignores the precise intersection-over-union (IOU) threshold, and a hit recall was counted if a ground truth box was hit by any detection result box. Accordingly, the precise positions of the cell nuclei are less important because the cells tend to form clumps in the Pap smear slides. Tan et al. [44] also used Faster RCNN architecture for cervical cancer cell detection in ThinPrep cytologic test (TCT) images scanned with a seamless slider at up to 400× and achieved AUC of 0.67. More recently, Ma et al. [42] proposed an improved Faster RCNN-FPN architecture for cervical cancer detection in cropped patches out of positive Pap smear images. They designed a lightweight booster consisting of a refinement and spatial-aware module, aimed to enhance feature details and spatial context information. 

As discussed above, the automated detection of cervical cancer cells in the cytology images is a multi-task process involving feature extraction, cell localization and classification. Although extensive research efforts in the past have made significant progress in automated classification and detection of the abnormal cervical cells, some bottle neck issues still remain open. Particularly relevant concerns include the following: (1) the abnormalities in the cancerous cells are subtle and complex; (2) Cells in the cervical Pap smear images exist in different sizes and their geometric shapes can be obscured due to clump formation and overlapping with artifacts; (3) The ROI pooling/aligning processes in the object detection algorithms tend to enhance the local receptive fields and lose global context information. 

Aimed at mitigating these potential limiting factors for more accurate detection of abnormal cells in cervical cytology images, in this study, we introduced two functional extensions for one of the top-performing object detectors, the Faster RCNN-FPN framework: One is the infusion of deformable convolution network (DCN) [47,48,49,50,51] in the last three stages of the bottom-up pathway of the FPN and the other is adding a global context aware (GCA) module [52] alongside the RPN. A unique advantage of the Faster RCNN-FPN architecture is its inherent multi-scale nature in feature extraction and can facilitate the cell detection of different sizes. Infusion of the deformable convolution layers into the later three stages of the FPN structure can explicitly learn the geometric offset information of the objects associated with shape transformations. We added a GCA module into the Faster RCNN-FPN to strengthen the spatial correlation between the background and foreground. In the GCA module, the feature pyramid and attention strategies are used for global feature extraction and feature refinement, respectively. We leveraged the extracted global features as attention maps for contextual modulation to improve the cell detection performance. Moreover, we utilized various conventional computer-vision techniques for imaging process to preprocess the negative image samples and tag some of the representative cells aimed to enhance the subtle feature differences between the negative and positive cancerous cells. We conducted extensive experiments using the dataset from the “Digital Human Body” (DHB) Vision Challenge (tianchi.aliyun.com/competition/entrance/231757/information (accessed on 1 February 2021)) with the proposed deformable and global context aware (DGCA) Faster RCNN-FPN architecture (hereon abbreviated as DGCA-RCNN). Besides the exhaustive test, we evaluated the performance of the proposed DGCA-RCNN framework and conducted systematic performance comparison with five other state-of-the-art object detectors including ATSS [53], RetinaNet [39], Faster RCNN-FPN [34], double-head [54], and Cascade RCNN [32]. We also carried out ablation analysis of the extensions in the DGCA-RCNN framework to assess their functional importance for the detector’s performance.

## 2. Materials and Methods

### 2.1. The Proposed Framework for Cervical Cancer Cell Detection

**Figure 1:**
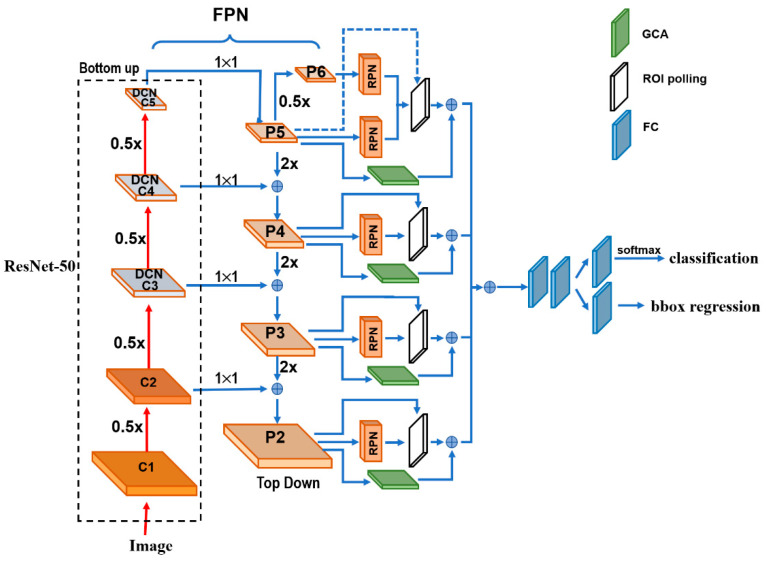
A schematic illustration of the proposed DGCA-RCNN architecture.

Figure 1 depicts the architecture details of the proposed DGCA-RCNN framework. It is based on the faster RCNN-FPN architecture consisting of image input, feature pyramid network (FPN) for feature extraction, feature map generation with region proposal network (RPN), classifier, and bonding box regressor. As shown in Figure 1, the FPN consists of a bottom-up and top-down pathways. The bottom-up pathway is a deep convolutional network hierarchy for feature extraction with successively decreasing spatial dimension. As more high-level features are extracted, the semantic value for each layer increases. The bottom layers (C1) are not included for object detection because of their limited semantic value and large dimension. They are in high resolution, require too much memory and can result in significant slowdown. The framework only uses the upper layers for detection and, therefore, performs much worse for smaller objects. However, FPN also provides a top-down pathway to construct higher resolution layers from a semantic rich layer using up-samplings. While the reconstructed layers are semantically strong, locations are not precise after all the down- and up-samplings. Therefore, lateral connections between the reconstructed layers and the corresponding feature maps are utilized to generate pixelwise addition to improve the localization precision. They also act as skip connections to facilitate training.

In this study, we used ResNet-50 to construct the bottom-up pathway. It composes of 5 convolution modules (C1–C5) and each has multiple convolution layers. From C1 to C5 the spatial dimension is reduced by half at each stage. We apply a 1 × 1 convolution filter to reduce the C5 channel depth to 256-d (P5). This becomes the first feature map layer used for object prediction. Along the top-down route, the previous layer is up-sampled by a factor of 2 using the nearest neighbor up-sampling method. The up-sampled P5 was added with the 1 × 1 filtered C4 pixel-wise to generate P4. The same process is repeated to generate P3 and P2. In this way, the FPN network utilizes its inherent multi-scale pyramidal hierarchy of deep convolutional networks to construct feature pyramids and make independent predictions at different levels (P2, P3, P4, P5 and P6) for multi-scale object detection. The RPN network loops through the different prediction levels and makes full use of the feature maps of different scales at 32 × 32, 64 × 64, 128 × 128, 256 × 256 and 512 × 512, respectively. Furthermore, three aspect ratios of 1:1, 1:2 and 2:1 are utilized at each level. The RPN is trainable from end-to-end to generate simultaneously bounding-box proposals and objectness scores at each position. These regions may contain target objects and are sent to the subsequent classifier network to produce the final classification and refined anchor positions. Since the same classifier/regressor model is shared among the region proposals from the feature map pyramid of different levels, the region of interest (ROI) pooling layer uses max pooling to convert each ROI from the RPN into a feature map of fixed size at 7 × 7.

### 2.2. Extensions to the Faster RCNN-FPN Architecture

The regular grids are used in the input feature maps for the convolution operations and CNNs are limited in modeling geometric transformations. Introducing deformable convolutional layers can add 2D offsets to the regular-grid sampling locations in the standard convolution and can facilitate the detection of the geometrically transformed objects without additional supervision [55]. Considering the geometrical variations of diploids in the cervical smear images, we replaced the last convolution layers in C3, C4 and C5 of the ResNet-50 [56] backbone by deformable convolutional layers in the proposed framework. To optimize the performance of the network and the efficiency of limited computation resource, the deformable convolution layers are usually implemented in C3–C5 of the FPN. The rationale is that the size of the feature maps in C1 and C2 are relatively large, computationally demanding, and not very sensitive to deformation effect. With the extension we can obtain the field offsets to model the various geometrical deformations of the cells on top of their ordinary feature maps. During training, both the convolutional kernels for generating the output features and the field offsets can be learned simultaneously. Therefore, we expect to improve the generality of the model in the aspects of scalability, geometry transformation and cell deformation.

As discussed above, ROI Pooling/Aligning is an indispensable process for the Faster RCNN-FPN architecture. It is used to rescale the object proposals cropped from the feature pyramid to generate a fixed-size feature map. However, these cropped feature maps of dominant local receptive fields possess very weak global context information. To alleviate this, we infused an off-the-shelf Global Context Aware (GCA) module to strengthen the spatial correlation between the background and foreground by fusing the global context information through attention mechanism. We leveraged the extracted global features as attention maps for contextual modulation to improve the cell detection. In the end, the GCA module also utilizes a max pooling to convert the global context features at different stages in the top-down route of FPN into the fixed size of 7 × 7 and merges them pixel-wise with the ROIs from the ROI pooling layer. The reference information from the background may help decide the spatial and category relationship between the targets and global contexts.

### 2.3. The Loss Functions

The loss function of the detection algorithm can be divided into two parts, one is the RPN loss, and the other is the loss of the detection network. To evaluate the RPN loss, we used the IOU metric which is defined as the ratio between the area of overlap and the area encompassed jointly by both the predicted bounding box and the ground-truth bounding box. Depending on the associated IOU each anchor is assigned a binary class label. If the IOU for a given anchor is >0.7 or <0.3, positive or negative label will be signed to the anchor, respectively. The detection model is optimized for a loss combining the two tasks: classification and localization. Therefore, the loss (*L*) function sums up the cost of classification (*L_cls_*) and bounding box prediction (*L_box_*):

(1) $$L=L_{cls}+L_{box}$$

(2) $$L\left(\left\{p_{i}\right\},\left\{t_{i}\right\}\right)=\frac{1}{N_{cls}}\underset{i}{\sum }L_{cls}\left(p_{i},\;p_{i}^{*}\right)+\frac{\lambda }{N_{box}}\underset{i}{\sum }p_{i}^{*}\cdot L_{1}^{smooth}\left(t_{i}-t_{i}^{*}\right)$$

where *L_cls_* is the log loss function over two classes (positive and negative), as we can easily translate a multi-class classification into a binary classification by predicting a sample being a target object versus not.

(3) Lcls(pi, pi*)=−pi*log pi −(1−pi*)log(1−pi)

The bounding box loss *L_box_* should measure the difference between $t_{i}$ and $t_{i}^{*}$ using a robust loss function. The smooth *L_1_* loss is adopted here as usual, and it is claimed to be less sensitive to outliers.

(4) $$L_{1}^{smooth}\left(x\right)=\{\begin{array}{c} 0.5x^{2}\;\;\;\;\;\;\;\;\;\;\;\;\;if\;\left|x\right|<1\\ \left|x\right|-0.5\;\;\;\;\;\;\;\;\;otherwise \end{array}$$

Other symbols’ definitions in the above equations are provided in Table 1.

### 2.4. The Dataset and Preprocessing Pipeline

For the study we used the cervical cytology image dataset from “Digital Human Body” (DHB) Vision Challenge-Intelligent Diagnosis of Cervical Cancer Risk provided by the Alibaba Cloud TianChi Company (https://tianchi.aliyun.com/competition/entrance/231757/information?scm=20140722.184.2.173 (accessed on 1 February 2021)). The original images were in KFB format and acquired with digital scanning under 20× magnification. Typical file size is in the range of 300–400 MB. The dataset contains 500 positive and 300 negative whole slide images (WSI) of 40,000 × 40,000. The positive WSIs have marked ROIs where the positions of 5414 lesions with abnormal squamous epithelial cells were labeled. The coordinate and size information for the ROIs were provided in an associated list document in json format. The abnormal squamous epithelial cells include mainly four types: atypical squamous cells (ASC-US) which cannot be clearly defined, low-grade intraepithelial lesions (LSIL), atypical squamous cells (ASC-H) which tend to have high intraepithelial cells, and high intraepithelial lesions (HIS). It is notable that there is no guarantee that there is no abnormal squamous epithelial cell outside the marked ROIs for the positive WSIs.

**Figure 2:**
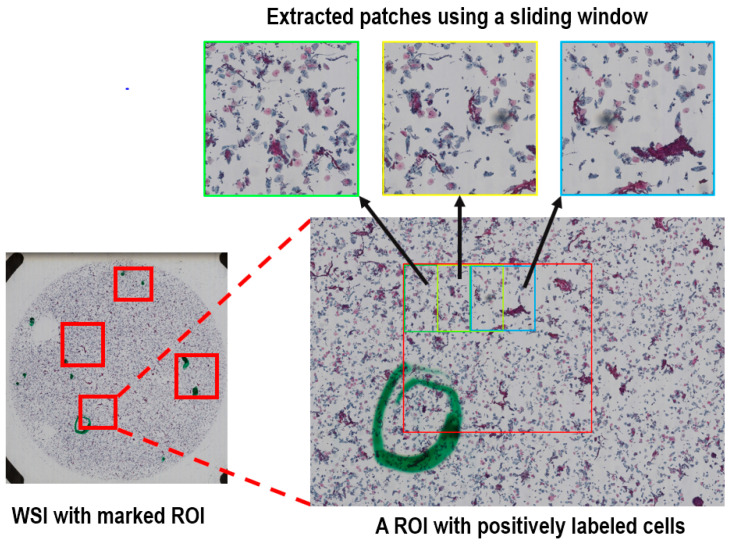
The preparation procedure for the positive WSIs to extract image patches of 2000 × 2000 out of the labeled ROIs using a sliding window with 50% overlap.

We utilized a library package provided by Ningbo Jiangfeng Konfoong Bioinformation Tech Co., Ltd (https://www.exporthub.com/eh-ningbo-konfoong-bioinformation-tech-co-ltd/ (accessed on 1 February 2021)) to convert from KFB to PNG format which has 3-channel RGB and 8-bit depth in each channel. Due to the limited GPU memory, the WSIs cannot be fed into the model directly and were cropped into smaller patches. As illustrated in Figure 2, for the training dataset we cropped all the marked ROIs in each positive WSI using a sliding window method to extract systematically patches of 2000 × 2000 out of each ROI. The overlap of the sliding window was 50% in both directions. On average, about 20 patches were generated per positive sample. 

For the negative samples without any specified ROI and the test dataset of the positive samples, we used the same sliding window method to crop the entire region within the radius of 20,000 voxels from the center of each WSI, because a statistical analysis of the marked ROIs for the positive samples showed that all positively labeled cells were within this range. There are rarely any cells at the edge of the images.

**Figure 3:**
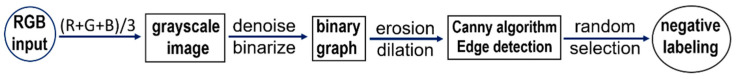
Summary of the traditional image preprocessing procedure used to select and label some of the cells in the negative image samples.

**Figure 4:**
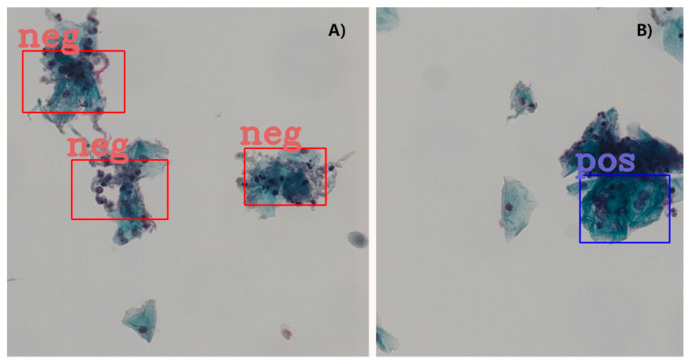
A zoomed display of an example set of the labeled cervical cells. (**A**) Labeled negative cells (normal), (**B**) labeled positive cells (cancerous).

To enhance the features in the negative image samples, we applied computer-vision techniques to label some of the negative cells. The negative tagging can improve the performance of the model and further reduce the false positive rate. As schematically illustrated in Figure 3, the procedure to label the negative cells included the following steps: Firstly, the RGB images were color averaged to generate grayscale images which were denoised using a series of filters. Then corrosion and dilation operations were applied to the binarized images before edge detection using the canny algorithm. Finally, for each of the preprocessed image patch a few cells of appropriate sizes were selected in a pseudo-random fashion and labeled as negative cells. The selection of the negative cells was carried by using a random number generator to attain an even distribution for the labeled negative cells after ranking the edge-detected negative cells according to their areas. For quality assurance the labeled negative samples were manually inspected to verify the exclusion of artifacts and background. An example set of the labeled cells (both negative and positive) are provided in Figure 4 in magnified display. The total number of the extracted image patches with negatively labeled cells was controlled to be the same as that for the positively labeled image patches (see Table 2).

To assess the performance of the trained model, we picked 80 positive and negative samples as the test dataset in a pseudo-random fashion. The rest of the dataset including both the positive and negative cases were used to train the proposed DGCA-RCNN model. The test dataset was also cropped by using the same sliding window described above to crop systematically each WSI within the radius of 20,000 voxels. The details of training and test datasets are summarized in Table 2.

We performed the standard data augmentation procedure by adding image rotations and adjustment of the overall brightness. Rotations were performed three times on each image with a step size of 90°. Rotation may slightly alter the image quality but should not change the abnormality or normality of the cells. The overall image brightness was changed by about ±20%. The slightly darkening or brightening the entire FOV should not affect the detection results. Changing the brightness of the images can replicate the clinical conditions of different image qualities and stain settings in preparing the slides. Zero-mean normalization to attain centralization of the input data was also implemented for each RGB channel. This can improve the generality of the model by reducing the interference from settings, such as the staining conditions and imaging equipment.

### 2.5. The Implementation and Training Procedure of the Models

We implement the DGCA-RCNN model using PyTorch (version 1.8.1). for the Python environment (version 3.5). The training and test of the model were carried out on a GPU server equipped with 4 NVIDIA TITAN X (Pascal) with 12 GB GPU memory. For comparison, based on the same backbone of ResNet50, we also implemented other five closely related the state-of-the-art object detection models developed in recent years including ATSS [53], RetinaNet [39], Cascade RCNN [32], Double head [54], and Faster RCNN-FPN [57]. We compared these models by strictly ruling out all the implementation inconsistencies between them.

Cross-validation procedure was implemented by further splitting the training-set into three folds and the model converged after about 12,000 iterations. To avoid over-fitting and prevent model selection bias, the cross-validation procedure was applied with a nested five-folds outer loop. After obtaining a score for each parameter set using the inner loop, we trained the model using the best parameter set from the three-folds inner loop on the whole data assigned to the inner loop without further manipulation of the parameter grid. The final model was selected according to the performance from the outer loop. We used the alternating training strategy to train the model, the RPN was first trained to generate the initial region proposals. The weights of the shared convolutional layers were initialized based on the pre-trained model using ImageNet. The other weights of the RPN were initialized randomly. The generated region proposals by the RPN were then used to train the Faster RCNN model. The weights of the shared convolutional layers were then initialized with the tuned weights by the RPN. The other Faster R-CNN weights were initialized randomly. When the weights for Faster RCNN and the shared layers were tuned, the tuned weights in the shared layers were again used to train the RPN, and the process repeats iteratively until convergency. In the initial stage, we used a warm-up strategy to attain learning stability and in the final stage we adopted a scheme of multi-step decay to improve convergency. In the first 500 iterations, the learning rate was linearly increased to 0.005. Then, the learning rate was kept flat in the next 7500 iterations. The ramp-down epochs were completed in three steps by reducing the learning rate by a factor of 10 in each step.

We also conducted ablation experiments with the proposed model to analyze the performance contributions from the different components, particularly the deformable convolution layers, GCA model and labeling of the negative samples.

### 2.6. Evaluation Metrics to Assess the Performance of the Models

When the IOU between the predicted and the ground truth bounding box exceeds a specified threshold value, it is considered as a correct detection. Depending on the specified confidence threshold, the detection results can have four possible types of outcomes: true positive (*TP*), false positive (*FP*), true negative (*TN*) and false negative (*FN*). Based on the detection results, the performances of the tested models are assessed using the following metrics:

Precision (*P*) is the model’s ability to identify only the relevant objects and is defined as the percentage of correct positive predictions among all positive detections:

(5) $$P=\frac{TP}{Total\;positive\;detection}=\frac{TP}{\left(TP+FP\right)}$$

Recall (*R*) is the model’s ability to detect all the relevant cases (also known as sensitivity) and defined as the percentage of the true positives among all positive ground truth cases:

(6) $$R=\frac{TP}{Total\;positive\;cancer\;cases}=\frac{TP}{\left(TP+FN\right)}$$

Both precision and recall metrics are important for object detection, it is, therefore, necessary to determine the precision–recall curve that shows the tradeoff between the precision and recall values for different thresholds. Furthermore, with the precision-recall curve the average precision (*AP*) can be estimated, which is a widely used metric to access the accuracy of an object detector. *AP* computes the average precision value for recall value over 0 to 1. The general definition for *AP* is to estimate the area under the precision-recall curve. That is

(7) $$AP=\int_{0}^{1}P\left(R\right)dR$$

In practical calculation of *AP*, the precision–recall curve, *P(R)*, is usually interpolated into multiple discrete points. For a set of queries (*n*), the mean average precision (*mAP*) is computed as follows:

(8) $$mAP=\frac{1}{n}\overset{k=n}{\underset{k=1}{\sum }}AP_{k}$$

where *k* is the query index in the set and *AP_k_* is the average precision for a given query *k*. In this study, we adopted the latest MS Common Objects in Context (COCO) method and a 101-point interpolated *AP* definition was used in the calculation for three different IOU thresholds. Besides the metrics outlined above, other metrics, such as specificity, sensitivity and area under curve (AUC) of the receiver operating characteristics (ROC) are also commonly used along for evaluating detection models. The ROC is a graph of the relationship between the true-positive rate (sensitivity is also known as recall) and the false-positive rate (1-specificity), which measures the effects of varying decision thresholds and accounts for all possible combinations of various correct and incorrect detections. Both *mAP* and AUC for the test set were computed for the following three IOU thresholds: 0.1, 0.3 and 0.5. Here the ROC calculation was based on the multiple cancer cells in the image.

## 3. Results

The number of parameters and frame per second (FPS) for the different models are summarized in Table 3.

Compared with the closely related model Faster RCNN-FPN, the proposed DGCA-RCNN model does not introduce significant overhead in terms of model complexity and computation burden. The number of parameters and time efficiency as indicated by FPS are quite comparable.

The results for the test dataset indicate that the proposed DGCA-RCNN framework can improve the detection performance for cervical abnormal cells at all tested 3 IOU thresholds compared with the other state-of-the-art models based on the same backbone ResNet50. As shown in Table 4, Table 5 and Table 6, the DGCA-RCNN model achieved the highest mAP and AUC at all IOU thresholds. Faster RCNN-FPN has the next best performance. Compared with the closely related model Faster RCNN-FPN, the mAP for the DGCA-RCNN model is boosted by 7–9% depending on the IOU threshold, while AUC is increased by 1–2%. As expected, the performance degrades with the increasing IOU thresholds and the corresponding mAP values are systematically reduced (see Table 4). A similar trend has also been observed for the AUC results (Table 5).

Table 7 shows the ablation results for the proposed model assessed at patch level for 3 different IOU thresholds. The mAP has been steadily boosted by adding the proposed extensions. Introducing deformable convolution layers improved mAP by 2.8–4.5%. The effect of introducing GCA module is quite inconsequential for mAP (0.1–0.6%), but nevertheless the effect is positive. The tagging of the negative image samples with computer vision techniques made significant improvement in mAP by 2.1–4.9%. To better understand this effect, we carefully compared the detection results with and without the tagging of the negative samples.

**Figure 5:**
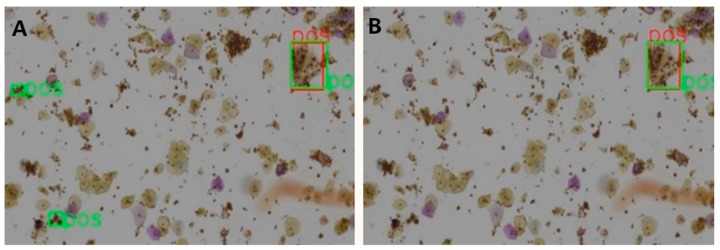
A zoomed display for the detection results without (**A**) and with (**B**) tagging of the negative image samples. The red-colored box indicates the positively labeled ground truth region, while the green-colored regions are detection results of the DGCA-RCNN framework.

A clear observation is that the negative tagging reduces false positive detections. Figure 5 shows such an example without (Figure 5A) and with (Figure 5B) the use of tagging the negative image samples. With the additional labeling information from the negative images, some subtle features of the negative cells appear to be enhanced and lead to reduced false positive detections, particularly in regions with clusters of nuclei.

## 4. Discussion

### 4.1. The Main Findings of the Study

The most important findings of our study are as follows: (1). There are currently few network architectures designed specifically for cervical cancer cell detection in Pap smear images [42,44,45,46]. Based on one of the currently top-performing detectors faster RCCN-FPN [57] and the characteristics of the cervical smear images, we proposed a novel DGCA-RCNN framework. Experimental results based on an openly accessible contest dataset of WSIs have demonstrated that the proposed architecture outperforms all other state-of-the-art models; (2) Tagging the negative samples with traditional computer-vision imaging techniques can reinforce the subtle and complex contrasts between the normal and abnormal cervical cells and reduce false positive detections; (3) Introducing deformable convolutional layers into the feature extraction pyramid network can improve scalability of the model and detection of deformed cells of various sizes; (4) Besides morphological and textural features, spatial context information can also be relevant for the detection of abnormal cells. Infusing context aware module into the detector can contribute positively to the performance of the framework.

### 4.2. Image Magnification and IOU Threshold

Detection of small cells in images is a challenging task due to the relatively small area with limited information. The Faster RCNN-FPN based architecture leverages the multiscale feature hierarchy to detect objects of various sizes [57]. For small objects there is little information left on the top-most feature map, which may compromise the detection of the small cells. FPN makes full use of the pyramidal feature maps by building a top-down pathway with lateral connections to the corresponding bottom-up feature maps, which significantly improves its detection accuracy compared with conventional detectors. Nevertheless, the features from the bottom levels have weak semantic information and could harm their representational capacity for small object recognition. As the system progressively reduces the input images to smaller feature maps at top levels, it retains little spatial information of small objects. Therefore, it is difficult to restore the lost details of small objects by up-sampling in the top-down route. Moreover, the combination of the low- and high-level features plays an important role in object detection. In the Faster RCNN-FPN based architectures feature maps of the 2 pathways are added in a simple fashion. Such direct feature combination may result in background clutter and semantic ambiguity.

In this study we used WSIs of 20× and the cells are relatively small. As discussed above, images with higher magnification are strongly favorable for the detection of cervical cancerous cells. A recent study [44] based on the Faster RCNN model used TCT images of 400× and achieved AUC = 0.67 with IOU threshold = 0.5. which is about 12% higher than the result obtained in this study for the similar model (Faster RCNN-FPN) and IOU. It is reasonable to attribute the difference to the magnification difference of the input WSIs. The basal diploids in cervical Pap smear images have large variations in size and tend to form clumps. Microscopic examinations are usually conducted at 200× to magnify the subtle feature differences between cells. Under low magnification as the dataset used in this study, the occupancy of the cells in an image patch is relatively small. The precise localization of the cells becomes quite challenging. A previous study suggested an interesting concept to alleviate this by counting any overlap between a ground truth box and the detection result as a true hit irrespective of the IOU threshold. In this study, we assessed the performance of the detector at three different IOU thresholds (0.1, 0.3 and 0.5). It is notable that mAP decreased from 0.505 to 0.445 when IOU was increased from 0.1 to 0.5. To facilitate accurate detection of cancerous cells, it is desirable to acquire sufficiently magnified image patches where the targeted cells occupy a significant fraction of the image, because details are required for the detector to automatically learn the subtle features and use them to detect the targeted cells. Partially obscured cells can be also identified if sufficient pixels are present. If the cells in the image patches appear too small, the detector’s performance may degrade to a point that the cells are misclassified or missed entirely. Very little research has been conducted to determine the performance limits for object detection algorithms when the object size is reduced [58,59]. Other limitations in image qualities [59], such as blurring, noise, contrast and lossy compression are even less known.

### 4.3. Limited Train Dataset and Unlabeled Data

The lack of train dataset is one of the main obstacles to develop machine learning methods for automated cervical cancer cell detection in cytology images because of privacy integrity and labeling cost issues [60]. A collection of at least hundreds of high-quality, well-curated digitized images of Pap smears and associated cell annotation results are required to train a machine learning model. The openly accessible Pap smears image database is currently quite limited and without balanced representation for cell types of pre-neoplastic alteration. As summarized in Table 8, the currently available datasets are mostly composed of cut-out cell images.

The Herlev dataset [27] is a widely used dataset for classification of cervical cancer cells. It consists of 917 single cell images, which have been categorized into seven pre-neoplastic lesions by cervical cytology professionals. The other dataset used for Pap smear image classification studies includes the SIPaKMeD database [61] consisting of 4049 annotated cells in 966 cut-out images. The cells have been manually classified into five cell types without pre-neoplastic alteration by expert cytopathologists. Some previous studies have relied on image collections of cut-out images [41,43] of clean images or positive samples [42]. Despite the seemingly good performance, these results are not so realistic and clinically relevant.

Extensive studies based on single and cut-out cells have significantly improved our understanding for the features of abnormal cervical cells [6,7,8,9,10,11,12,13,14,15,16,17,18]. As illustrated in Figure 4, the negative and positive cervical cancerous cells have a wide range of subtle feature differences, including optical density, size, shape, texture and contextual information. The most informative features for the cervical cell classification are the nucleus/cytoplasm ratio, brightness of nuclei and cytoplasm, and longest linear dimension and area of nuclei. However, the classification of single cell images and detection of cervical cancerous cells in WSIs are quite different tasks. The latter is more challenging because it requires not only classification of the recognized abnormal lesions but also the identification and localization of the abnormal cervical cells in WSIs. Unlike the cut-out single cell images, WSIs from cervical cancer screening tests have a broad diversity of neoplastic lesions and many other challenges, including image quality issues discussed above, overlapping cells and inflammatory cells. Moreover, the datasets to train a detection model need to provide the ground-truth locations and size of the cancerous lesions and such datasets are rarely available. To the best of our knowledge, the DHB dataset used in the current study is the only cervical Pap smear WSI dataset labeled for cancer cell detection. Besides the relatively low magnification issues discussed above, there are number of important limitation factors associated with the dataset, which can significantly influence the detection performance. Firstly, the lesions were not exhaustively labeled and there is no guarantee that the cells outside the tagged regions are all normal. This means that false positive detections outside the tagged regions can be true positive, and the detection performance can, therefore, be underestimated. Secondly, the occupancy of the labeled regions was only a small fraction (<1 ppm) of the WSI images. This implies that the relative populations between the abnormal and normal cells are substantially out of balance (in the order of 10^4^) and the clinical relevance of the detection algorithm should focus on reducing false negative detections. During model training, we attempted to balance the positive and negative labeled samples (see Table 2). Therefore, there are substantial fraction of cells are unlabeled both in the negative and positive samples. Considering the potentially large number and wide diversity of object instances in Pap Smear WSIs, it is inherently a challenging task to constitute complete exhaustive annotations. The missing annotations can be problematic, as the standard cross-entropy loss employed to train the model implicitly treats the unlabeled regions as background. Any unlabeled object (without a bounding box) can result in a confusing learning signal [62]. This can be addressed by removing the assumption that unlabeled regions must be the background.

## 5. Conclusions

In this study, we proposed a novel DGCA-RCNN framework for the detection of abnormal cervical cells in Pap smear images. We extended the Faster RCNN-FPN model by introducing deformable convolution layers into FPN to improve scalability and adding a GCA module alongside RPN to enhance the spatial context information. The results from extensive experiments have demonstrated that the proposed model has significantly improved mAP and AUC for the detection of abnormal cells in Pap smear WSIs and outperform other state-of-art detectors. When combined with tagging of the negative image samples using computer-vision techniques, 6–9% increase in mAP has been achieved. The main limiting factors include WSI magnification and adequate labeling. The proposed DGCA-RCNN model has great potential to become a clinically useful AI tool for automated detection of cervical cancer cells in Pap smear images.

## Figures and Tables

**Table 1:** Definitions of the symbols used in the loss functions.

Symbol	Explanation
*i*	The index of anchors in a mini batch.
*p_i_*	Predicted probability of anchor *i* being an object.
$p_{i}^{*}$	Ground truth label (binary) of whether anchor *i* is an object.
*t_i_*	Predicted four-parameter coordinates for anchor *i*.
$t_{i}^{*}$	Ground truth coordinates.
*N_cls_*	Normalization term, set to be mini-batch size (~256).
*N_box_*	Normalization term, set to the number of anchor locations,
λ	A balancing parameter, set to be ~10, so that both *L_cls_* and *L_box_* terms are roughly equally weighted.

**Table 2:** The number of WSIs and cropped patches for the training and test datasets.

Category	Positive		Negative	
	WSI	Patch	WSI	Patch
Training set	420	7801	220	7801
Test set	80	104,972	80	122,560

**Table 3:** The number of parameters and FPS for the different models.

Model	Parameters	FPS
ATSS [53]	31.89 MB	3.5
RetinaNet [39]	36.6 MB	3.7
Cascade RCNN [32]	68.93 MB	3.1
Double head [54]	47.3 MB	2.1
Faster RCNN-FPN [57]	41.53 MB	3.6
DGCA-RCNN *	42.11 MB	3.3

* The proposed framework of the present study.

**Table 4:** Comparison of the mAP at three different IOU levels for the proposed DGCA-RCNN framework with other state-of the-art methods for object detection.

Model	mAP@0.1	mAP@0.3	mAP@0.5
ATSS [53]	0.362	0.354	0.329
RetinaNet [39]	0.374	0.363	0.341
Cascade R-CNN [32]	0.385	0.38	0.355
Double head [54]	0.388	0.382	0.355
Faster RCNN-FPN [34]	0.415	0.394	0.371
DGCA-RCNN *	0.505	0.486	0.445

* The proposed framework of the present study.

**Table 5:** The AUC results for the ROC curves at three different IOU thresholds.

Model	ROC @0.1	ROC @0.3	ROC @0.5
ATSS [53]	0.603	0.562	0.503
RetinaNet [39]	0.633	0.584	0.526
Cascade RCNN [32]	0.653	0.609	0.548
Double head [54]	0.522	0.489	0.446
Faster RCNN-FPN [34]	0.652	0.610	0.557
DGCA-RCNN *	0.670	0.625	0.569

* The proposed framework of the present study.

**Table 6:** Comparison of the true positive and false positive cancerous cell numbers as detected by the different models at three different IOU thresholds.

Model	Total	TP Cells			FP Cells		
		@0.1	@0.3	@0.5	@0.1	@0.3	@0.5
ATSS [53]	17,850	779	721	633	17,071	17,129	17,217
RetinaNet [39]	28,200	765	698	624	27,435	27,502	27,576
Cascade RCNN [32]	28,102	786	728	651	27,316	27,374	27,451
Double head [54]	10,235	691	638	575	9544	9597	9660
Faster RCNN-FPN [34]	27,814	780	728	661	27,034	27,086	27,153
DGCA-RCNN *	19,426	808	750	681	18,618	18,676	18,745

* The proposed framework of the present study.

**Table 7:** The ablation results for the proposed DGCA-RCNN model at three different IOU thresholds. The contributions associated with DCN, GCA and tagging of negative samples were analyzed.

Scale	DCN	GCA	Negative	mAP_0.1_	mAP_0.3_	mAP_0.5_
Patch				0.415	0.394	0.371
	√			0.461	0.439	0.399
	√	√		0.467	0.445	0.396
	√	√	√	0.505	0.486	0.445

**Table 8:** Summary of the openly accessible Pap smear image datasets in the literature.

Property	CRIC Cervix [60]	Herlev [27]	SIPakMed [61]	DHB
Images (*n*)	400	917	966	500/300
Cells per image	multiple	1	multiple	many
Image type	Cut-out cluster	Single cell	Isolated cells	WSI
Image size	1376 × 1020	variable	2048 × 1536	40,000 × 40,000
Resolution	0.228 μm/pixel	0.201 μm/pixel	unknown	20×
Classified Cells	11,534	917	4049	5414
Validation	3 cytologists	2 cytologists	cytologists	Cytologists
source	database.cric.com.br	mde-lab.aegean.gr	cs.uoi.gr	*

* tianchi.aliyun.com/competition/entrance/231757/information (accessed on 1 Feburary 2021).

## Data Availability

The image data used in the study are openly accessible at the following site: https://tianchi.aliyun.com/competition/entrance/231757/introduction (accessed on 1 Feburary 2021).
